# Antimicrobial Properties and Cytocompatibility of PLGA/Ag Nanocomposites

**DOI:** 10.3390/ma9010037

**Published:** 2016-01-11

**Authors:** Mariangela Scavone, Ilaria Armentano, Elena Fortunati, Francesco Cristofaro, Samantha Mattioli, Luigi Torre, Jose M. Kenny, Marcello Imbriani, Carla Renata Arciola, Livia Visai

**Affiliations:** 1 Unité Mixte de Recherche (UMR) S949, Inserm, Strasbourg 67000, France; Mariangela.Scavone@efs.sante.fr; 2 Etablissement Français du Sang-Alsace (EFS-Alsace), Strasbourg 67000, France; 3 Fédération de Médecine Translationnelle de Strasbourg (FMTS), Strasbourg 67000, France; 4 Université de Strasbourg, Strasbourg 67000, France; 5 Materials Engineering Center, UdR INSTM, University of Perugia, Terni 05100, Italy; ilaria.armentano@unipg.it (I.A.); elena.fortunati@unipg.it (E.F.); samantha.mattioli@unipg.it (S.M.); luigi.torre@unipg.it (L.T.); jose.kenny@unipg.it (J.M.K.); 6 Department of Molecular Medicine, INSTM UdR of Pavia, Biochemistry Unit, “A Castellani”, Viale Taramelli, 3/b-27100 Pavia, Center for Health Technologies (C.H.T.), University of Pavia, Pavia 27100, Italy; francesco.cristofaro01@universitadipavia.it; 7 Department of Public Health, Experimental Medicine and Forensics, University of Pavia, Pavia 27100, Italy; marcello.imbriani@unipv.it or marcello.imbriani@fsm.it; 8 Department of Occupational Medicine, Toxicology and Environmental Risks, S. Maugeri Foundation, IRCCS Pavia 27100, Italy; 9 Research Unit on Implant Infections, Rizzoli Orthopaedic Institute, and DIMES, University of Bologna, Via di Barbiano 1/10-40136, Bologna, Italy

**Keywords:** Ag nanocomposites, cell cytocompatibility, antibacterial activity

## Abstract

The purpose of this study was to investigate the antimicrobial properties of multifunctional nanocomposites based on poly(dl-Lactide-co-Glycolide) (PLGA) and increasing concentration of silver (Ag) nanoparticles and their effects on cell viability for biomedical applications. PLGA nanocomposite films, produced by solvent casting with 1 wt%, 3 wt% and 7 wt% of Ag nanoparticles were investigated and surface properties were characterized by atomic force microscopy and contact angle measurements. Antibacterial tests were performed using an *Escherichia coli* RB and *Staphylococcus aureus* 8325-4 strains. The cell viability and morphology were performed with a murine fibroblast cell line (L929) and a human osteosarcoma cell line (SAOS-2) by cell viability assay and electron microscopy observations. Matrix protein secretion and deposition were also quantified by enzyme-linked immunosorbent assay (ELISA). The results suggest that the PLGA film morphology can be modified introducing a small percentage of silver nanoparticles, which induce the onset of porous round-like microstructures and also affect the wettability. The PLGA/Ag films having silver nanoparticles of more than 3 wt% showed antibacterial effects against *E. coli* and *S. aureus*. Furthermore, silver-containing PLGA films displayed also a good cytocompatibility when assayed with L929 and SAOS-2 cells; indicating the PLGA/3Ag nanocomposite film as a promising candidate for tissue engineering applications.

## 1. Introduction

Polymeric nanocomposites have emerged in the last two decades as an efficient strategy for the improvement of material properties in order to create novel polymer-based systems with peculiar structural and functional characteristics [1]. Generally, polymer nanocomposites are the result of the combination of polymers and inorganic/organic fillers at the nanometer scale, taking advantage of the inherent high surface area/volume ratio of nano-materials [2,3], surface engineering being a tool for modifying and adapting materials to specific biological applications [4,5].

Silver (Ag) has been known to have a disinfectant effect and has found applications in traditional medicine, for the treatment of burns and chronic wounds [6]. The antimicrobial property of silver is related to the amount of silver and to the rate of silver release. Silver in its metallic state is inert, but it reacts with the moisture in the skin and the fluid of the wound and becomes ionized. The ionized silver is highly reactive, as it binds to tissue proteins and brings structural changes in the bacterial cell wall and nuclear membrane, leading to cell distortion and death. Silver also binds to bacterial DNA and RNA, to protein sulfhydryl groups, eventually inhibiting bacterial replication [7,8,9]. Recently, aspects such as the interaction of silver at a molecular level and at a cellular level, the literature dealing with nanomaterials from which we can learn and get clues on the functioning of silver at all levels, and finally studies on the biocompatibility of silver, have been reported [10].

Concerning silver nanoparticles, some authors have provided a comprehensive view on the mechanism of action, production, applications in the medical field, and the health and environmental concerns that are allegedly caused by their usage [11]. The silver nanoparticles show an efficient antimicrobial property compared to other systems due to their extremely large surface area, which provides better contact with microorganisms. The nanoparticles get attached to the cell membrane and also penetrate inside the bacteria. The nanoparticle diameter is a key point in cell penetration accordingly to the cell type. The nanoparticles release silver ions in the bacterial cells, which enhance their bactericidal activity [12,13,14]. Silver nanoparticles have drawn considerable interest for their capability to release silver ions in a controlled manner, which, in turn, leads to a powerful antibacterial activity [15,16,17]. However, their use has been limited by difficulties associated with handling and processing nanoparticles. In fact, they are easily aggregated because of their high surface free energy, and they can be oxidized in air by the action of humidity. Embedding nano-sized metals into biodegradable polymer matrices represents a valid solution to these stabilization problems and permits a controlled antibacterial effect [18].

Aliphatic polyesters play a very important role in the field of biodegradable materials and now their nanocomposites are attracting growing interest from researchers. Poly(dl-Lactide-co-Glycolide) (PLGA) is a biocompatible and biodegradable polymer approved by Food and Drug Administration (FDA) [19]. The development of PLGA/Ag nanocomposites opens new challenges in the field of bio-medicine, yielding biocompatible polymers with modulated morphological, surface roughness (the changes to the surface nanostructures induced by the introduction of specific amount of nanoparticles) and antibacterial properties [20].

The silver nanoparticles with their unique chemical and physical properties are proving an alternative for the development of new antibacterial agents [21,22,23]. However, with the advent of silver nanoparticles and its major use as an antimicrobial agent, much experimental trials are needed to understand the toxicity and the effects on living cells. There are some questions which need to be addressed, such as the exact mechanism of interaction of silver nanoparticles with the bacterial cells, how the surface area of nanoparticles influences its killing activity, the use of animal models and clinical studies to reach a better understanding of the antimicrobial efficiency of silver dressings, the toxicity if any of the silver dressings, *etc*. In this regard, data related to genotoxicity and cytotoxicity of silver nanoparticles, in order to better understand the possible applications of these nanomaterials in a safe manner, have been reported [24,25].

In this paper, PLGA/Ag nanocomposite films were developed by solvent cast processing and characterized in terms of surface morphology, contact angle and antibacterial properties. The functional role of Ag nanoparticles was also investigated by studying their effects on a murine fibroblast cell line (L929) and on a human osteosarcoma cell line (SAOS-2).

## 2. Results

### 2.1. Film Characterization

**Figure 1:**
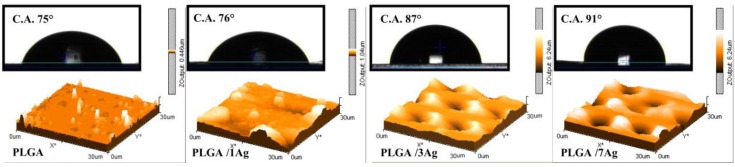
Atomic force microscopy and water contact angle images of PLGA, PLGA/1Ag, PLGA/3Ag and PLGA/7Ag films.

Figure 1 shows atomic force microscopy and contact angle images of PLGA, PLGA/1Ag, PLGA/3Ag and PLGA/7Ag nanocomposites developed by using a solvent casting procedure in CHCl_3_ at different content of Ag nanoparticles. AFM images of the upper surface, exposed to air during the solvent casting process underline a superficial and circular porous microstructure with a pore diameter of 13.0 ± 2.0 μm, 10.0 ± 0.5 μm, 10.5 ± 1.0 μm and a depth of 1.0 ± 0.5 μm, 2.5 ± 0.5 μm, 2.5 ± 0.5 μm, for PLGA/1Ag, PLGA/3Ag and PLGA/7Ag respectively, while a flat configuration was observed in the pristine polymer films [22,23].

Water contact angle (CA) images are reported in Figure 1. Neat PLGA film shows a contact angle of 75°, which indicates a moderate hydrophilicity. With the increase of Ag content, the contact angle increases from 75° for PLGA pristine polymer, to 76° for PLGA/1Ag, to 87° for PLGA/3Ag, and to 91° for PLGA/7Ag. Thus, silver nanoparticles reduce the PLGA hydrophilicity, bringing the polymer up to the hydrophobicity.

### 2.2. In Vitro Antimicrobial Properties of Ag Nanoparticles

To evaluate antimicrobial properties of Ag nanoparticles, bacterial adhesion and antibacterial activity were performed using *E. coli* RB and *S. aureus* 8325-4 strains.

**Figure 2:**
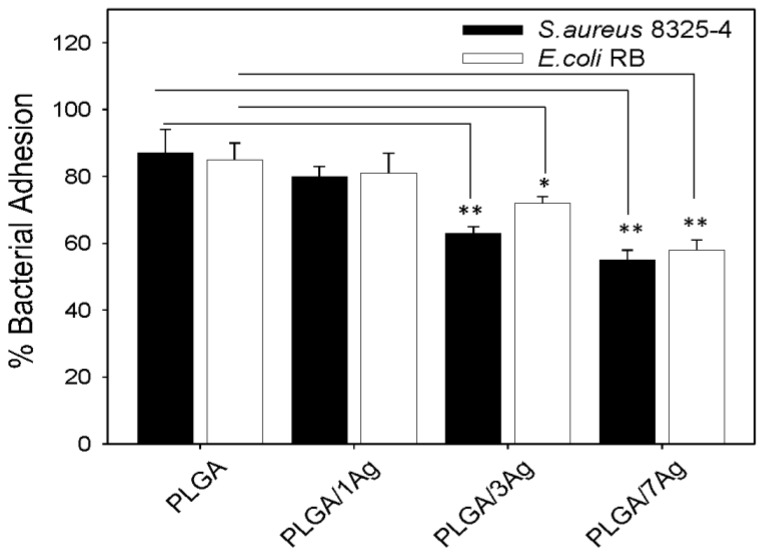
Bacterial adhesion to PLGA and PLGA/Ag nanocomposite films. *S. aureus* and *E. coli* cell adhesion to PLGA and PLGA/Ag was determined as colony forming units (CFU/mL) after 3 h incubation at 37 °C. Data are expressed as percentage of the ratios between CFU of bacteria adherent to PLGA to CFU of bacteria adherent to 24-well flat-bottom sterile polystyrene microplates. The values represented are the means of the results of each sample performed in duplicate and repeated in three separated experiments. Error bars indicate standard errors of the means. The statistical significance was indicated as follows: * *p* < 0.05 and ** *p* < 0.01.

Studies on cell adhesion showed a reduction in adhesion for both bacterial strains to all samples if compared to a neat PLGA system (Figure 2). On PLGA/Ag samples, the reduction of bacterial adhesion was dependent on increasing content of Ag nanoparticles, varying from 20% up to 45% (*p* < 0.05). The results were the highest on PLGA/7Ag films for both bacterial strains but slightly lower for *S. aureus* cells if compared to *E. coli* cells.

**Figure 3:**
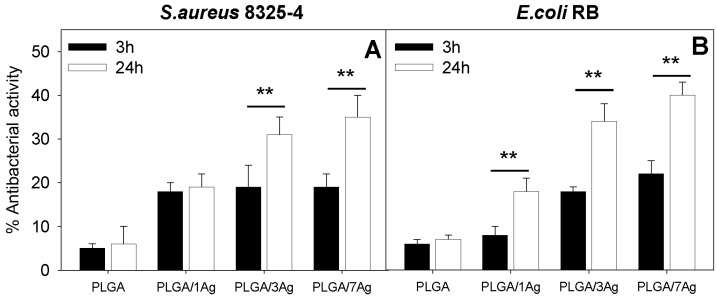
Antibacterial activity of PLGA and PLGA/Ag nanocomposite films. Surviving fractions of *S. aureus* (**A**) and *E. coli* (**B**) cells to the indicated PLGA films were determined as CFU/mL after 3 and 24 h incubation times. Data are expressed as percentage of the ratios between CFU of bacteria grown on PLGA films to CFU of bacteria grown in 24-well flat-bottom sterile polystyrene plates. The values represented are the means of the results of each sample performed in duplicate and in three separate experiments. Error bars indicate standard errors of the means. The statistical significance was indicated as follows: ** *p* < 0.01.

In Figure 3, the antibacterial activity exerted by neat PLGA and PLGA/Ag nanocomposites with increasing concentrations of Ag (1, 3 and 7 wt%) on *S. aureus* (Figure 3A) and *E. coli* (Figure 3B) growth is reported. As expected, the survival of both *S. aureus* and *E. coli* cells was particularly high on neat PLGA at 3 h and 24 h with no statistical significance (*p* > 0.05). The antibacterial effect, as inhibition of the growth of both bacterial species, was Ag dose-dependent, showing the greatest value with PLGA/7Ag nanocomposite films either at 3 and 24 h (*p* < 0.05). The reduction in growth of both bacterial species on each PLGA/Ag nanocomposite film was statistically significant if compared with neat PLGA (*p* < 0.05). Interestingly, after 24 h, the antibacterial effect on both bacterial cells was considerably enhanced on respect to the 3 h incubation time on PLGA/3Ag and PLGA/7Ag nanocomposite films (*p* < 0.05). In particular, the antibacterial effect was slightly higher for *E. coli* cells if compared to *S. aureus* cells.

### 2.3. In Vitro Effect of Ag Nanoparticles on Cell Viability and Morphology

**Figure 4:**
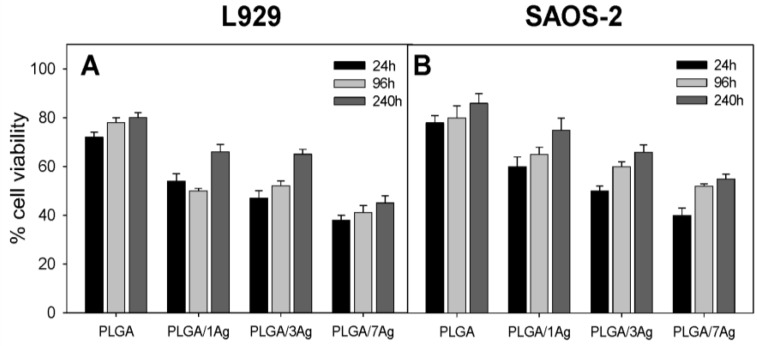
L929 and SAOS-2 cell viability. At 24 h, 96 h and 240 h of culture, cell viability was determined by the MTT assay performed on PLGA, PLGA/1Ag, PLGA/3Ag and PLGA/7Ag films. Panel (**A**) shows L929 cells viability whereas panel (**B**) represents SAOS-2 cells viability cultured in osteogenic medium. The error bars represent the standard deviations.

In order to correlate the previously indicated antimicrobial properties of PLGA/Ag nanocomposite films with cell viability, the MTT test was performed during the indicated culture times (Figure 4). The results for L929 (Figure 4A) and SAOS-2 (Figure 4B) cells are reported. On days 1, 4, and at the end of the culture period (10 d), the average cell viability of PLGA samples was in the 76%–86% range for both cell types without statistically significant differences (*p* > 0.05). These results are quite important since they show that PLGA itself does not affect both cell types in the adhesion and proliferation process. On the contrary, for both cell types and the indicated cultures times, the average cell viability of PLGA/Ag nanocomposites was significantly at lower range if compared to PLGA samples: 55%–76% for PLGA/1Ag, 50%–70% for PLGA/3Ag and 38%–48% for PLGA/7Ag, respectively (*p* < 0.05). The cell viability was Ag nanoparticles dose-dependent, showing the lowest cell viability for both cell types on the PLGA/7Ag nanocomposite films. In particular, at the end of the culture period, an increment in cell viability for all PLGA/Ag nanocomposites was observed.

**Figure 5:**
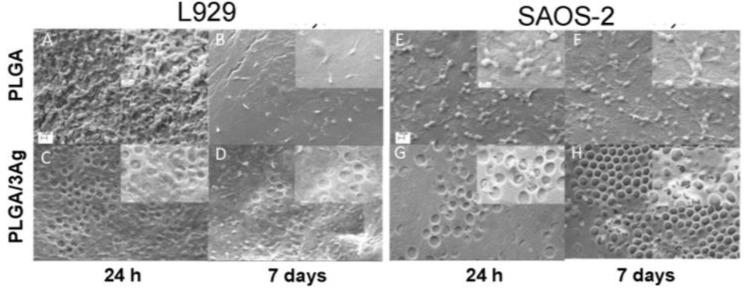
Representative SEM images of the cell cultured on PLGA, and PLGA/3Ag films. Both cell types, L929 (**A**–**D**) or SAOS-2 (**E**–**H**), were seeded and cultivated for 24 h (**A**,**C**,**E**,**G**) and seven days (**B**,**D**,**F**,**H**), on PLGA (**A**,**B**,**E**,**F**) and PLGA/3Ag (**C**,**D**,**G**,**H**), respectively, and then fixed for SEM observations (×1000 magnification). Both fibroblasts (panels (**A**,**B**)) and osteoblasts (panels (**E**,**F**)) coated the PLGA surface forming a homogenous layer at 24 h and day 7: individual cells were no longer discernable over the surface and above this layer some cells exhibited a round shape (insert of panels (**A**,**B**,**E**,**F**) at ×3000 magnification); on the PLGA/3Ag films (**C**,**D**,**G**,**H**), fibroblasts and osteoblasts did not form a homogenous layer at 24 h or day 7 even if some cells could be observed into the holes of the materials (insert of panels (**C**,**D**,**G**,**H**) at ×3000 magnification). Scale bars represent 10 μm (**A**–**H**) and 2 μm (all the inserts), respectively.

The morphology and the shape of the cells seeded on the surfaces of PLGA and PLGA/3Ag samples were examined by scanning electron microscopy. Figure 5 showed that L929 fibroblasts and SAOS-2 osteoblasts were well spread on the PLGA and on the rough surfaces of PLGA/3Ag either at a shorter or longer incubation time. In particular, on PLGA film fibroblasts and osteoblasts formed a homogenous layer (Figure 5A,B,E,F) whereas on PLGA/3Ag samples were not completely coated by both cell types (Figure 5C,D,G,H) even if some cells were observed in the inner part of the formed pores due to surface structure (Figure 5, inserts of panels C,D,G,H). SEM observations confirmed cell viability (Figure 4) on PLGA and PLGA/3Ag films.

### 2.4. In Vitro Effect of Ag Nanoparticles on Extracellular Matrix (ECM) Deposition by L929 and SAOS-2 Cells

To characterize the extracellular matrix deposition by both cell types, the total protein concentration was determined after 10 d of cell culture. Protein extracted from samples cultivated with L929 cells gave the following results: (462 ± 9) μg/ mL on neat PLGA, (370 ± 8) μg/mL on the PLGA/1Ag, (370 ± 5) μg/mL on the PLGA/3Ag and (302 ± 6) μg/mL on PLGA/7Ag nanocomposite films. Similar results were obtained for samples cultivated with SAOS-2 cell in osteogenic medium: (404 ± 15) μg/mL on neat PLGA, (379 ± 10) μg/mL on the PLGA/1Ag, (321 ± 9) μg/mL on the PLGA/3Ag and (262 ± 7) μg/mL on PLGA/7Ag nanocomposite films.

In order to evaluate the amount of the extracellular matrix constituents produced on all types of nanocomposites, an ELISA assay of the extracted extracellular matrix was performed. For samples cultivated with L929 cells, fibronectin (Fn) and collagen (Col) were evaluated as protein constituents of the extracellular matrix deposited (Table 1), whereas for SAOS-2 cells, all the principal constituents of bone ECMs were determined (Table 2).

**Table 1:** Normalized amount of extracellular matrix constituents secreted and deposited by L929 cells cultivated for 10 d on poly(dl-Lactide-co-Glycolide) (PLGA) and PLGA/silver (Ag) samples (pg/cell × sample).

Matrix Protein Deposition after 10 d of Cell Culture Expressed as pg/(Cell × Sample)				
Samples	Fibronectin (Fn)	Ratio Fn PLGA Containing Ag/PLGA	Type I Collagen (Col)	Ratio Col PLGA Containing Ag/PLGA
**PLGA/PLGA**	5.4 ± 0.2	1	8.4 ± 0.2	1
**PLGA/1Ag**	4.5 ± 0.1	0.83	7.3 ± 0.3	0.86
**PLGA/3Ag**	4.2 ± 0.3	0.77	6.6 ± 0.4	0.75
**PLGA/7Ag**	3.4 ± 0.1	0.62	5.1 ± 0.1	0.61

**Table 2:** Normalized amount of extracellular matrix constituents secreted and deposited by a human osteosarcoma cell line (SAOS-2) cells cultivated for 10 d on PLGA and PLGA/Ag samples (pg/Cell × Sample).

Matrix Protein Deposition after 10 d of Cell Culture Expressed as pg/(cell × well)							
Samples	PLGA	PLGA/1Ag	Ratio PLGA/1Ag and PLGA	PLGA/3Ag	Ratio PLGA/3Ag and PLGA	PLGA/7Ag	Ratio PLGA/7Ag and PLGA
**Alkaline Phosphatase**	2.9 ± 0.2	2.9 ± 0.5	1.00	3.0 ± 0.1	1.03	2.2 ± 0.2	0.75
**Decorin**	14.0 ± 0.1	15.0 ± 0.3	1.07	11.2 ± 1.2	0.80	10.5 ± 0.1	0.80
**Fibronectin**	0.9 ± 2.8	0.5 ± 0.1	0.53	0.3 ± 1.2	0.33	0.49 ± 0.01	0.55
**Osteocalcin**	1.68 ± 0.1	0.72 ± 0.02	0.42	0.9 ± 1.2	0.53	1.0 ± 0.2	0.59
**Osteonectin**	2.5 ± 0.3	1.4 ± 0.2	0.55	2.5 ± 0.3	1.02	1.71 ± 0.02	0.69
**Osteopontin**	15.1 ± 0.3	9.0 ± 0.4	0.59	8.8 ± 1.1	0.58	4.5 ± 1.2	0.29
**Type-I collagen**	20.0 ± 1.0	17.5 ± 1.4	0.87	14.5 ± 1.9	0.72	12.7 ± 1.1	0.63
**Type-III collagen**	2.7 ± 0.1	3.7 ± 0.1	1.36	3.0 ± 0.2	1.09	1.90 ± 0.11	0.69

Table 1 reports the normalized amount of fibronectin and collagen secreted and deposited by L929 cells cultivated for 10 d on PLGA and PLGA/Ag samples (pg/cell × sample). At the end of the culture period, in comparison with the PLGA films, the deposition of Fn and type I collagen on all the PLGA/Ag samples was considerably reduced as a function of the increased content of Ag nanoparticles.

To evaluate the amount of ECM constituents produced by SAOS-2 cells on PLGA and PLGA/Ag nanocomposite films, an ECM extraction was performed on day 10. At the end of the culture period, the detection of bone proteins showed some differences in comparison to PLGA (Table 2). The deposition of the majority of bone matrix proteins was reduced on PLGA/7Ag nanocomposites if compared to PLGA, PLGA/1Ag and PLGA/3Ag samples. PLGA/1Ag and PLGA/3Ag nanocomposite films showed quite similar results for most proteins but different if compared to PLGA (*p* < 0.05). In particular, in comparison to PLGA neat film, the content of type I collagen, fibronectin, osteocalcin and osteopontin on PLGA/1Ag and PLGA/3Ag was slightly lower (Table 2); no significant difference on PLGA/1Ag and PLGA/3Ag was observed for the content of alkaline phosphatase (ALP), decorin, osteonectin and type III collagen (Table 2). These data are encouraging since the presence of an increasing quantity of Ag up to 3% in PLGA seems to slightly affect the bone matrix deposition.

**Figure 6:**
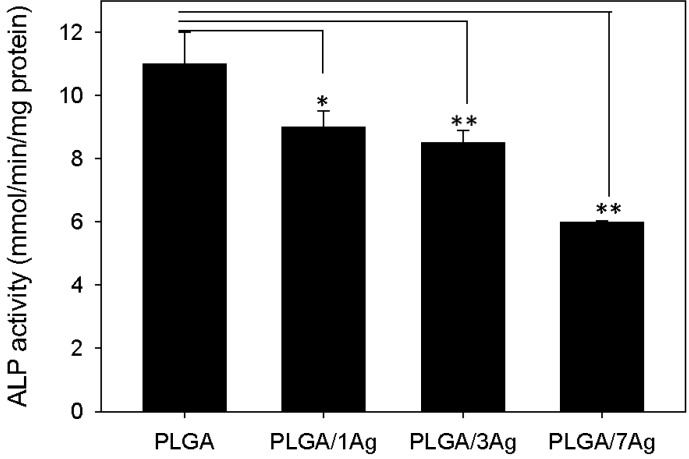
ALP activity of SAOS-2 cells. Cells were seeded on PLGA, PLGA/1Ag, PLGA/3Ag and PLGA/7Ag films and cultured in osteogenic medium for 10 d. ALP activity was determined colourimetrically, corrected for the protein content measured with the BCA Protein Assay Kit and expressed as millimoles of p-nitrophenol produced per min per mg of protein. Bars express the mean values ± SEM of results from three experiments (* *p* < 0.05; ** *p* < 0.01).

Figure 6 shows ALP activity determined on all PLGA samples at the end of the culture period with SAOS-2 cells. The level of ALP activity was consistently lower on PLGA/Ag nanocomposite films than on the PLGA (*p* < 0.05), even if it was quite similar for PLGA/1Ag and PLGA/3Ag samples.

## 3. Discussion

Nanocomposites based on silver nanoparticles were successfully developed on PLGA as polymer matrix with different Ag nanoparticle content and a homogeneous nanoparticle dispersion, as previously demonstrated by confocal microscopy [26].

The AFM images reveal the formation of ring-like microstructures on PLGA nanocomposites that is the result of two phenomena: the chloroform evaporation and the presence of silver nanoparticles. Low concentrations of silver nanoparticles are able to induce surface morphological changes in the polymer matrix and affect the surface nanocomposite wettability and roughness; all these aspects can influence the bacterial adhesion as well as the cell biocompatibility on the nanocomposite surface [27,28,29]. Highly hydrophobic behavior is associated to surface roughness increase induced by the presence of silver nanoparticles. Contact angle, in fact, depends on several factors such as surface preparation, roughness, chemical and physical configuration that can influence biomaterial properties.

The bacterial adhesion and antibacterial activity were tested on PLGA film and PLGA/Ag samples containing increasing content of Ag nanoparticles using an *S. aureus* and an *E. coli* strain as previously reported [27]. The main difference in respect to the previous study [24,25,26] is the addition of the PLGA/3Ag nanocomposite film in the bacterial assays. The results are quite interesting showing that on a PLGA/Ag films having silver nanoparticles around 3 wt%, the adhesion percentage is reduced for both bacterial strains but resulted quite similarly to the sample containing 7 wt% of Ag. Bacterial adhesion is a very complicated process that is affected by many factors, including material wettability and surface chemical composition. Consequently, the reduction in bacteria adhesion that characterized the PLGA/3Ag and PLGA/7Ag films can be attributed to the increased roughness properties induced by the addition of the silver nanoparticles and confirmed by morphological characterization and wettability studies.

The antibacterial activity of PLGA/Ag nanocomposites was slightly more effective on *E. coli* than on *S. aureus* cells as previously reported [22]. Again, the antibacterial activity was time-dependent showing better results at longer incubation times (24 h). This may be ascribed to the release, even if quite slowly and slight, of silver ions (Ag^+^) from the PLGA matrix. Ag^+^ ions are known to bind strongly to electron donor groups in biological molecules containing sulfur, oxygen, or nitrogen causing defects in the bacteria cell wall so that cell contents are lost, leading to the death of the bacterial cells [30,31]. Biocompatibility has been considered as “the ability of a material to perform with an appropriate host response in a specific application” [32], taking into account the interactivity between the biomaterial and the host. Considering the chemical-physical properties of these biomaterials, an application in tissue engineering is suggested. To this purpose, the biocompatibility studies were performed with L929 and SAOS-2 cells. Mouse connective tissue fibroblasts L929 are a standard model for biocompatibility testing, since they are easy to cultivate and because of their favorable doubling time of about 24 h. The use of L929 cells is acceptable since they are widely used for cytotoxicity studies. Furthermore, to test the potential application of these biomaterials as a coating of medical devices, the SAOS-2 cell line was selected as it exhibits several fundamental osteoblast characteristics [33] and represents a widely used model for *in vitro* osteoblast study. In contrast to the other cell lines, these osteoblasts can be grown indefinitely, and they exhibit unique osteoinductive activity [33,34,35,36].

Poly(dl-Lactide-co-Glycolide) (PLGA) is known as a biocompatible and biodegradable polymer approved by Food and Drug Administration (FDA) [19,20] but the addition of Ag nanoparticles may vary its biocompatibility. Silver nanoparticles are known to be cytotoxic either *in vitro* either *in vivo* [23]. As expected, PLGA material showed a high level of adhesion and proliferation for both cell types, independently of the incubation times. On the contrary, the increasing concentrations of Ag nanoparticles caused a significant decrement in viability of both cell types if compared to PLGA neat film. Interestingly, the reduction in cell viability was much higher at 24 h that at 96 h or longer incubation time. This result can be related to the release of Ag^+^ that is reduced at longer incubation times as previously indicated [27]. Fibroblasts are the most common cells of connective tissue in animals. They synthesize the extracellular matrix (*i.e.*, Fn) and collagen, the structural framework (stroma) for animal tissues, that plays a critical role in wound healing. Fn, as the prototype of cell adhesive and bioactive matrix proteins, has been shown to regulate cell growth and shape, cytoskeletal organization, differentiation, migration, and apoptosis of almost all tissue cells [37,38,39]. About collagen, it is known as the major insoluble fibrous protein in the extracellular matrix and in connective tissue [40]. Furthermore, 80%–90% of the collagen in the body consists of types I–III, even if more than 23 types of collagen are recognized [40]. For a biocompatibility study, it becomes important to evaluate the fibroblast ECM secretion because of cell direct interaction with PLGA and PLGA/Ag nanocomposite samples. The deposition of fibronectin and collagen was reduced on PLGA/Ag nanocomposites if compared to PLGA samples. The reduction in protein content expression was related to the increment in Ag nanoparticles, showing the PLGA/7Ag sample as the poorest. Interestingly, the Fn and collagen protein content was quite similar for PLGA/1Ag and PLGA/3Ag. This may suggest that the PLGA/Ag films having silver nanoparticles between 1 and 3 wt% are more biocompatible than PLGA/7Ag sample. It has been reported that an increase in bone formation depends on an increase in extracellular matrix synthesis [41]. Therefore, we evaluated the deposition of specific bone matrix components by ELISA assay. The results normalized to the number of cells per each Ag nanocomposite films showed a decreasing content value for all tested proteins in comparison with PLGA samples. The decrement in protein content is acceptable, and it is related to the increase in Ag nanoparticles showing the lowest value for PLGA/7Ag film. However, the reduction in protein deposition was quite similar for PLGA/1Ag and PLGA/3Ag. Interestingly, the reduction was detected for type I collagen, fibronectin, osteocalcin, and osteopontin but was much higher on PLGA/7Ag film if compared to PLGA. All of these ECM proteins are organic components of bone and are implicated in bone formation and remodeling: their reduction needs to be considered in the evaluation process of a biomaterial for bone tissue engineering application.

The ALP protein content was almost similar for the PLGA, PLGA/1Ag and PLGA/3Ag, whereas it was quite low for PLGA/7Ag. ALP is known as an important component in hard tissue formation, highly expressed in mineralized tissue cells. Osteoblast cells grown with ascorbic acid sequentially express osteoblastic marker proteins such as alkaline phosphatase and then form a mineralized extracellular matrix (ECM) because of osteoblastic differentiation. Its mechanism of action is not completely known, but it appears both to promote the local concentration of inorganic phosphate, a mineralization promoter, and to decrease the concentration of extracellular pyrophosphate, an inhibitor of mineral formation [42,43].

These results may suggest that the osteoblasts on PLGA/1Ag and PLGA/3Ag films are differentiated as those on PLGA samples and have already started to promote bone ECM deposition. However, the PLGA/3Ag nanocomposite film proved to be a better biomaterial since it combines good antibacterial and biocompatibility properties. Even if some other parameters such as calcium deposition and bone gene transcription need to be evaluated, we may suggest this biomaterial as the promising candidate for a future tissue engineering approach.

## 4. Experimental Section

### 4.1. Materials

Poly(dl-Lactide-co-Glycolide) (PLGA) (Inherent Viscosity (I.V.) 0.95–1.20 dL/g) ether terminated, an amorphous copolymer with a 50/50 ratio (PLA/PGA) with glass transition temperature of 45 °C, was purchased from Absorbable Polymers-Lactel (Durect Corporation, Birmingham, UK). Commercial silver nanopowder, P203, was supplied by Cima NanoTech (Corporate Headquarters Saint Paul, MN, USA) and characterized as previously reported [26].

### 4.2. Development and Characterization of Ag Nanocomposite Films

PLGA nanocomposite samples were developed by solvent casting in chloroform (CHCl_3_). Silver nanopaticle content were selected at 1 wt%, 3 wt% and 7 wt% with respect to the polymer matrix. Neat PLGA films were obtained dissolving polymer granules in chloroform at a fixed concentration (10 wt%/v) and by using a magnetic stirring at room temperature (RT) until a complete polymer dissolution. Nanocomposite films were developed by dispersing silver nanoparticles in CHCl_3_, as previously reported [26,27], by means of sonication treatment for 5 h, in order to avoid aggregation and Ag cluster presence, and to enhance the interaction with the biodegradable matrix. Thereafter, the polymer was added to solutions and the suspensions were magnetically stirred until they were completely dissolved. The obtained dispersions were cast on a Teflon surface, allowing the solvent to evaporate over 24 h and leaving films of rectangular shape (0.3 mm in thickness) [26,27]. Samples were further dried for 48 h in vacuum at room temperature (RT). Resulting films were designed as PLGA, PLGA/1Ag, PLGA/3Ag and PLGA/7Ag.

Atomic force microscopy (AFM, Easy Scan, Nanosurf, Liestal, Switzerland), was used to evaluate the topography induced by the introduction of Ag nanoparticles. AFM images were recorded in tapping mode at room temperature in air.

Static contact angles (CA) were measured by FTA 1000 Analyser, USA at room temperature, in order to investigate the nanocomposite surface wettability as a function of the Ag loading. The CA was assessed by the sessile drop method in air with drop shape analysis of 20 μL deionized water. Five measurements were averaged to obtain the data.

### 4.3. In Vitro Bacterial Assays on PLGA and PLGA/Ag Nanocomposites

#### 4.3.1. Bacterial Strains and Culture Conditions

The microorganisms used in this study were *Escherichia coli* RB (*E. coli* RB) and *Staphylococcus aureus* 8325-4 (*S. aureus* 8325-4). *E. coli* RB was an isolate provided by the “Zooprofilattico Institute of Pavia”, Italy, whereas *S. aureus* 8325-4 was a gift from Timothy J. Foster (Department of Microbiology, Dublin, Ireland). *E. coli* RB was routinely grown in Luria Bertani Broth (LB) (Difco, Detroit, MI, USA) and *S. aureus* 8325-4 in Brian Heart Infusion (BHI) (Difco) overnight under aerobic conditions at 37 °C using a shaker incubator (New Brunswick Scientific Co., Edison, NJ, USA). These cultures, used as source for the experiments, were reduced at a final density of 1 × 10^10^ cells/mL as determined by comparing the OD600 of the sample with a standard curve relating OD600 to cell number.

#### 4.3.2. Bacterial Adhesion Assay

To evaluate the adhesion of *E. coli* or *S. aureus* cells to each sample of PLGA and PLGA/Ag films, an overnight suspension of both strains were washed with sterile PB saline buffer (PBS: 137 mM NaCl, 2.7 mM KCl, 4.3 mM Na_2_HPO_4_, 1.4 mM KH_2_PO_4_, pH 7.4) and an aliquot of 200 μL (1 × 10^6^ cells) was seeded on the sample and incubated for 3 h. Briefly, as previously reported [44], loosely adhering bacteria were removed by gently washing the samples with PBS, whereas the bacterial cells tightly adherent to the surfaces were prepared for SEM analysis (data not shown). Three samples of each experimental condition were used for Total Viable Count (TVC) estimation. The samples with the adherent bacterial cells were dispersed into 1 mL sterile Ringer solution (Oxoid, Italy) by vortex for 5 min. The cleaned surfaces were stained and observed to ensure complete recovery of cells. Serial dilutions of the bacterial cell suspensions were prepared and 0.1 mL of each dilution was deposited onto BHI or LB agar (Bacto agar, Difco, Franklin Lakes, NJ, USA) plates, depending on the bacterial strains. The plates were incubated for 24 h–48 h at 37 °C and the number of colonies counted. Mean TVC values were calculated for each sample and the percent viability was calculated by setting the bacterial cells grown onto 24 tissue culture plates (TCP) wells equal to 100%.

#### 4.3.3. Antibacterial Activity

To examine the antimicrobial activity of each sample of PLGA and PLGA/Ag films, 200 µL (5 × 10^3^ cells) of an overnight diluted suspension of *E. coli* or *S. aureus* cells was added and incubated for 24 h, respectively [45]. Wells used as controls were incubated with cell suspension for 24 h. At the end of the incubation time, bacterial suspension was serially diluted, and plated on the LB (*E. coli*) or BHI (*S. aureus*) agar plates, respectively. The plates were incubated for 24 h–48 h at 37 °C. The percent viability was calculated by setting the bacterial cells grown onto 24 TCP wells equal to 100%.

### 4.4. In Vitro Cell Assays on PLGA and PLGA/Ag Nanocomposites

#### 4.4.1. Cell Types and Culture Growth Conditions

The murine fibroblast cell line L929 and the human osteosarcoma cell line SAOS-2 were obtained from the American Type Culture Collection (ATCC, Manassas, VA, USA). L929 cells were cultured in RPMI-1640 medium (Cambrex Bio Science, Baltimore, MD, USA), supplemented with 10% (*v*/*v*) fetal bovine serum, 1% l-glutamine, 1% sodium pyruvate and 1% penicillin/streptomycin (Sigma-Aldrich, St. Louis, MO, USA). SAOS-2 cells were cultured in McCoy’s 5A modified medium with l-glutamine and HEPES (Cambrex Bio Science, Baltimore, MD, USA), supplemented with 15% foetal bovine serum, 2% sodium pyruvate, 1% antibiotics, 10^−8^ M dexamethasone, and 10 mM β-glycerophosphate (Sigma-Aldrich) [40]. The osteogenic factor β-glycerophosphate was used at 10 mM concentration as previously reported in the literature [45,46]. Ascorbic acid, another osteogenic supplement, is a component of McCoy’s 5A modified medium. Both cell types were cultured at 37 °C with 5% CO_2_, routinely trypsinized after confluency, counted, and seeded onto each PLGA and PLGA nanocomposites films.

#### 4.4.2. Cell Seeding and Culture

PLGA and PLGA/Ag films were sterilized by UV treatment. Each film (diameter size 1.5 cm) was placed inside a standard 24-well-plate and were washed first with sterile distilled water, after with 0.9% NaCl sterile solution and finally with culture medium. A cell suspension (200 μL) containing 2 × 10^4^ (L929) and 1 × 105 (SAOS-2) cells was added onto the top of each film and, after 0.5 h, 0.8 mL of culture medium was added to cover the samples and incubated for the times indicated. The culture medium was changed every three days.

#### 4.4.3. 3-(4,5-Dimethylthiazole-2-yl)-2,5-Diphenyl Tetrazolium Bromide Test

To evaluate the mitochondrial activity of seeded cells of both types, that is, the cell viability on the PLGA and PLGA/Ag nanocomposite films during the culture period, a test with 3-(4,5-dimethylthiazole-2-yl)-2,5-diphenyl tetrazolium bromide (MTT) (Sigma-Aldrich), was performed on days 1, 4, and 10 (end of the culture period) as previously reported [39]. Aliquots of 200 μL were sampled, and a microplate reader (BioRad Laboratories, Hercules, CA, USA) measured the related absorbance values at 570 nm (with reference wavelength at 690 nm). A standard curve of cell viability was used to express the results as a percentage.

#### 4.4.4. DNA Content

At the end of incubation, the cells were lysed by a freeze-thaw method in sterile deionised distilled water. The released DNA content was evaluated with a fluorometric DNA quantification kit (PicoGreen; Molecular Probes, Eugene, OR, USA). A DNA standard curve [45] obtained from a known amount of fibroblasts and osteoblasts was used to express the results as cell number per each PLGA and PLGA/Ag nanocomposite films.

#### 4.4.5. Scanning Electron Microscopy (SEM) Analysis

At 24 h and 7 d of incubation with L929 and SAOS-2 cells, the PLGA and PLGA/3Ag samples were fixed with 2.5% (*v*/*v*) glutaraldehyde solution in 0.1 M Na-cacodylate buffer (pH 7.2) for 1 h at 4 °C, washed with Na-cacodylate buffer, and then dehydrated at room temperature in an ethanol gradient series up to 100%. The samples were kept in 100% ethanol for 15 min, and then critical point-dried with CO_2_. The specimens were sputter coated with gold and observed at ×1000 and ×3000 magnification respectively with a Zeiss EVO-MA10 scanning electron microscope (Carl Zeiss, Oberkochen, Germany) [46].

#### 4.4.6. Purified Proteins

Decorin, type-I collagen and fibronectin were purified as described previously [47]; osteocalcin was acquired from Biomedical Technologies (Stoughton, MA, USA), osteopontin and osteonectin were obtained from Assay Designs (Ann Arbor, MI, USA); type-III collagen and alkaline phosphatase were purchased from Sigma-Aldrich.

#### 4.4.7. Polyclonal Antisera

Dr. Larry W. Fisher (National Institutes of Health, Bethesda, MD, USA) provided us with the rabbit polyclonal anti type-I and type-III collagen, anti-decorin, anti-osteopontin, anti-osteocalcin, anti-osteonectin, and anti-alkaline phosphatase. Polyclonal antibody against human fibronectin (HFn) was produced as previously described [40].

#### 4.4.8. Extraction of the Extracellular Matrix Proteins from the Cultured Scaffolds and ELISA Assay

At the end of the culture period, in order to evaluate the amount of the extracellular matrix constituents on the film surface, the samples were washed extensively with sterile PBS to remove the culture medium, and then incubated for 24 h at 37 °C with 1 mL of sterile sample buffer (20 mM Tris-HCl, 4 M GuHCl, 10 mM EDTA, 0.066% (*w*/*v*) SDS, pH 8.0). At the end of the incubation period, sample buffer aliquots were removed, and then the films were centrifuged at 4000 rpm for 15 min in order to collect the sample buffer entrapped inside the materials. The total protein concentration in both culture systems was evaluated with the BCA Protein Assay Kit (Pierce Biotechnology, Rockford, IL, USA). After matrix extraction, the PLGA and PLGA/Ag nanocomposite films were incubated, once again, for 24 h at 37 °C with 1 mL sterile sample buffer, and no protein was detected. Calibration curves to measure type-I and type-III collagens, decorin, osteopontin, osteocalcin, osteonectin, fibronectin and alkaline phosphatase were performed as previously described [41]. In order to measure the extracellular matrix amount of each protein, an ELISA assay was performed as previously reported [42]. We have taken into consideration that an underestimation of the absolute protein deposition is possible because the sample buffer, used for matrix extraction, contained sodium dodecyl sulphate (SDS), which may interfere with protein adsorption during the ELISA assay. The amount of extracellular matrix constituents from all PLGA and PLGA/Ag nanocomposite films was expressed as pg/(cell per film).

#### 4.4.9. Alkaline Phosphatase (ALP) Activity

ALP activity was determined using a colorimetric end point assay [41]. The assay measures the conversion of the colorless substrate *p*-nitrophenol phosphate (PNPP) by the enzyme ALP to the yellow product p-nitrophenol, where the rate of color change corresponds to the amount of enzyme present in solution. The test was performed as previously described [42] on cells cultured with/without osteogenic factors. Samples were run in triplicate and compared against the calibration curve of p-nitrophenol standards. The enzyme activity was expressed as micromoles of p-nitrophenol produced per min per mg of enzyme.

### 4.5. Statistical Methods

Continuous data were expressed as means and SD. Both bacterial strains like as L929 and SAOS-2 cells grown in 24-TCP were used as the reference (100%). For bacterial adhesion, antibacterial activity and cell viability, two-group comparisons (PLGA and each PLGA/Ag nanocomposites) were performed by Student’s *t*-test. All analyses were performed using GraphPad Prism 4.0 (Graph Pad Software Inc., San Diego, CA, USA). Two-tailed *p* values < 0.05 were considered statistically significant.

## 5. Conclusions

Nanocomposite films based on PLGA polymer matrix and Ag nanoparticles were successfully developed by solvent casting process. Ag nanoparticles induce antibacterial properties on PLGA based samples, and maintained good biocompatibility properties. However, the PLGA/3Ag nanocomposite film proved to be a better biomaterial since it combines good antibacterial and biocompatibility properties. Even if some other parameters such as calcium deposition and bone gene transcription need to be evaluated, we may suggest this biomaterial as a promising candidate for a future tissue engineering approach. In summary, to our knowledge, this is the first work that has combined the nanocomposite approach by using silver nanoparticles to develop polymeric surfaces with tunable chemical, physical, antibacterial properties and cell biocompatibility studies.

## References

[1] Armentano I., Dottori M., Fortunati E., Mattioli S., Kenny J.M. (2010). Biodegradable polymer matrix nanocomposites for tissue engineering: A review. Polym. Degrad. Stab..

[2] Tjong S.C. (2006). Structural and mechanical properties of polymer nanocomposites. Mater. Sci. Eng. R..

[3] Schaefer D.W., Justice R.S. (2007). How nano are nanocomposites?. Macromolecules.

[4] Legeay G., Poncin-Epaillard F., Arciola C.R. (2006). New surfaces with hydrophilic/hydrophobic characteristics in relation to (no) bioadhesion. Int. J. Artif. Organs.

[5] Arciola C.R., Caramazza R., Pizzoferrato A. (1994). *In vitro* adhesion of *Staphylococcus epidermidis* on heparin-surface-modified intraocular lenses. J. Cataract. Refract. Surg..

[6] Rai M., Yadav A., Gade A. (2009). Silver nanoparticles as a new generation of antimicrobials. Biotechnol. Adv..

[7] Landsdown A.B.G. (2002). Silver I: Its antibacterial properties and mechanism of action. J. Wound Care.

[8] Castellano J.J., Shafii S.M., Ko F., Donate G., Wright T.E., Mannari R.J., Payne W.G., Smith D.J., Robson M.C. (2007). Comparative evaluation of silver-containing antimicrobial dressings and drugs. Int. Wound J..

[9] Taglietti A., Arciola C.R., D’Agostino A., Dacarro G., Montanaro L., Campoccia D., Cucca L., Vercellino M., Poggi A., Pallavicini P. (2014). Antibiofilm activity of a monolayer of silver nanoparticles anchored to an amino-silanized glass surface. Biomaterials.

[10] Eckhardt S., Brunetto P.S., Gagnon J., Priebe M., Giese B., Fromm K.M. (2013). Nanobio silver: Its interactions with peptides and bacteria, and its uses in medicine. Chem. Rev..

[11] Prabhu S., Poulose E.K. (2012). Silver nanoparticles: Mechanism of antimicrobial action, synthesis, medical applications, and toxicity effects. Int. Nano Lett..

[12] Panacek A., Kvitek L., Prucek R., Kolar M., Vecerova R., Pizurova N., Sharma V.K., Nevecna T., Zboril R. (2006). Silver colloid nanoparticles: Synthesis, characterization, and their antibacterial activity. J. Phys. Chem. B.

[13] Morones J.R., Elechiguerra J.L., Camacho A., Holt K., Kouri J.B., Ramirez J.T., Yacaman M.J. (2005). The bactericidal effect of silver nanoparticles. Nanotechnology.

[14] Baker C., Pradhan A., Pakstis L., Pochan D.J., Shah S.I. (2005). Synthesis and antibacterial properties of silver nanoparticles. J. Nanosci. Technol..

[15] Falletta E., Bonini M., Fratini E., lo Nostro A., Pesavento G., Becheri A., lo Nostro P., Canton P., Baglioni P. (2008). Clusters of poly(acrylates) and silver nanoparticles: Structure and applications for antimicrobial fabrics. J. Phys. Chem. C.

[16] Evanoff D.D., Chumanov G. (2005). Synthesis and optical properties of silver nanoparticles and arrays. Chem. Phys. Chem..

[17] Campoccia D., Montanaro L., Arciola C.R. (2013). A review of the biomaterials technologies for infection-resistant surfaces. Biomaterials.

[18] Lee J.Y., Nagahata J.L.R., Horiuchi S. (2006). Effect of metal nanoparticles on thermal stabilization of polymer/metal nanocomposites prepared by a one-step dry process. Polymer.

[19] Ma P.X. (2004). Scaffold for Tissue Engineering. Mater. Today.

[20] Park G.E., Pattison M.A., Park K., Webster T.J. (2005). Accelerated chondrocyte functions on NaOH-treated PLGA scaffolds. Biomaterials.

[21] Campoccia D., Montanaro L., Arciola C.R. (2013). A review of the clinical implications of anti-infective biomaterials and infection-resistant surfaces. Biomaterials.

[22] Fortunati E., Latterini L., Rinaldi S., Kenny J.M., Armentano I. (2011). PLGA/Ag nanocomposites: *In vitro* degradation study and silver ion release. J. Mater. Sci. Mater. Med..

[23] Armentano I., Kenny J.M., Armentano I., Kenny J.M. (2013). Silver Nanoparticles: Synthesis, Uses and Health Concerns.

[24] De Lima R., Seabra A.B., Durán N. (2012). Silver nanoparticles: A brief review of cytotoxicity and genotoxicity of chemically and biogenically synthesized nanoparticles. J. Appl. Toxicol..

[25] Hadrupa V., Lamb H.R. (2014). Oral toxicity of silver ions, silver nanoparticles and colloidal silver—A review. Regul. Toxicol. Pharmacol..

[26] Armentano I., Fortunati E., Latterini L., Rinaldi S., Saino E., Visai L., Elisei F., Kenny J.M. (2010). Biodegradable PLGA matrix nanocomposite with silver nanoparticles: Material properties and bacteria activity. J. Nanostruct. Polym. Nanocomp..

[27] Fortunati E., Mattioli S., Visai L., Imbriani M., Fierro J.L.G., Kenny J.M., Armentano I. (2013). Combined effects of Ag nano-particles and oxygen plasma treatment on PLGA morphological, chemical, and antibacterial properties. Biomacromolecules.

[28] Agarwal A., Weis T.L., Schurr M.J., Faith N.G., Czuprynski C.J., McAnulty J.F., Murphy C.J., Abbott N.L. (2010). Surfaces modified with nanometer-thick silver-impregnated polymeric films that kill bacteria but support growth of mammalian cells. Biomaterials.

[29] An Y.H., Friedman R.J. (1998). Concise review of mechanisms of bacterial adhesion to biomaterial surfaces. J. Biomed. Mater. Res..

[30] Damm C., Münstedt H., Rösch A. (2008). The Antimicrobial Efficacy of Polyamide 6/Silver-Nano and Microcomposites. Mater. Chem. Phys..

[31] Monteiro D.R., Gorup L.F., Takamiya A.S., Ruvollo-Filho A.C., de Camargo E.R., Barbosa D.B. (2009). The growing importance of materials that prevent microbial adhesion: Antimicrobial effect of medical devices containing silver. Int. J. Antimicrob. Agents.

[32] Williams D.F. (1999). The Williams Dictionary of Biomaterials.

[33] Anderson H.C., Hsu H.H.T., Raval P., Reynold P.R., Gurley D.J., Aguilera M.X., Davis L.S., Moylan P.E. (1998). Bone-inducing agent in Saos-2 cell extracts and secretions. Cells Mater..

[34] Anderson H.C., Reynolds P.R., Hsu H.H., Missana L., Masuhara K., Moylan P.E., Roach H.I. (2002). Selective synthesis of bone morphogenetic proteins-1, -3, -4 and bone sialoprotein may be important for osteoinduction by Saos-2 cells. J. Bone Miner. Metab..

[35] Anderson H.C., Sugamoto K., Morris D.C., Hsu H.H., Hunt T. (1992). Bone-inducing agent(BIA) from cultured human Saos-2 osteosarcoma cells. Bone Miner..

[36] Anderson H.C., Hsu H.H., Raval P., Hunt T.R., Schwappach J.R., Morris D.C., Schneider D.J. (1995). The mechanism of bone induction and bone healing by human osteosarcoma cell extracts. Clin. Orthop. Relat. Res..

[37] Akiyama S.K., Yamada K.M. (1987). Fibronectin. Adv. Enzymol. Relat. Areas Mol. Biol..

[38] Humphries M.J., Obara M., Olden K., Yamada K.M. (1989). Role of fibronectin in adhesion, migration, and metastasis. Cancer Investig..

[39] Larsen M., Artym V.V., Green J.A., Yamada K.M. (2006). The matrix reorganized: Extracellular matrix remodeling and integrin signaling. Curr. Opin. Cell Biol..

[40] Burgeson R.E., Nimni M.E. (1992). Collagen types. Molecular structure and tissue distribution. Clin. Orthop. Relat. Res..

[41] Manolagas S.C. (2000). Birth and death of bone cells: Basic regulatory mechanisms and implications for the pathogenesis and treatment of osteoporosis. Endocr. Rev..

[42] Torii Y., Hitomifl K., Yamagishi Y., Tsukagoshi N. (1996). Demonstration of alkaline phosphatase participation in the mineralization of osteoblasts by antisense RNA approach. Cell Biol. Int..

[43] Zimmermann R., Pfuch A., Horn K., Weisser J., Heft A., Roder M., Linke R., Schnabelrauch M., Schimanski A. (2011). An approach to create silver containing antibacterial coatings by use of atmospheric pressure plasma chemical vapour deposition (APCVD) and combustion chemical vapour deposition (CCVD) in an economic way. Plasma Process. Polym..

[44] Petrini P., Arciola C.R., Pezzali I., Bozzini S., Montanaro L., Tanzi M.C., Speziale P., Visai L. (2006). Antibacterial activity of zinc modified titanium oxide surface. Int. J. Artif. Organs.

[45] Saino E., Maliardi V., Quartarone E., Fassina L., Benedetti L., de Angelis M.G., Mustarelli P., Facchini A., Visai L. (2010). *In vitro* enhancement of SAOS-2 cell calcified matrix deposition onto radio frequency magnetron sputtered bioglass-coated titanium scaffolds. Tissue Eng. A.

[46] Saino E., Grandi S., Quartarone E., Maliardi V., Galli D., Bloise N., Fassina L., de Angelis M.G., Mustarelli P., Imbriani M. (2011). *In vitro* calcified matrix deposition by human osteoblasts onto a zinc-containing bioactive glass. Eur. Cell Mater..

[47] Bloise N., Ceccarelli G., Minzioni P., Vercellino M., Benedetti L., de Angelis M.G., Imbriani M., Visai L. (2013). Investigation of low-level laser therapy potentiality on proliferation and differentiation of human osteoblast-like cells in the absence/presence of osteogenic factors. J. Biomed. Opt..

